# Variability in brain network model dynamics: comparison of neural mass models and empirical connectivity datasets in The Virtual Brain

**DOI:** 10.1186/1471-2202-14-S1-P197

**Published:** 2013-07-08

**Authors:** M Marmaduke Woodman, Viktor K Jirsa

**Affiliations:** 1 Institut de Neuroscience des Systemes UMR 1106, Aix-Marseille University, Marseille, 13005, France

## 

The so-called rest state of the human brain has revealed a rich repertoire of dynamic patterns as viewed through neuroimaging modalities [1]. Much of this richness is thought to come from inherent multistable structure of cortical activity, yet only recently have theoretical studies on the origin of these dynamics begun to dissect the relations between corticocortical connectivity, local neural dynamics and the global, whole brain dynamics [2,3]. In the case of realistic connectivities, the delays due to finite conduction delays between regions presents a major roadblock for analytic approaches, and simulation becomes the main workhorse for understanding cortical dynamics. In this work, we contribute to the theory of such rest state cortical activity by exploring the variability of spatiotemporal pattern formation in brain networks as a function of neural mass model dynamics, connectivity and intersubject variability. We find that the neural mass model chosen for network modeling can reshape the stability structure of cortical dynamics. Additionally, it is shown that the fine structure of the stability of the dynamics depends on the joint distribution of connection weights and delays. Lastly we also show that manipulations of the connectivity delays can reshape the functional connectivity on the longer time scale of several seconds or minutes. In general, we attempt to layout the first steps towards an atlas on the modeled cortical dynamics of the human brain.
